# Development of the Crohn's disease digestive damage score, the Lémann score

**DOI:** 10.1002/ibd.21506

**Published:** 2010-11-28

**Authors:** Benjamin Pariente, Jacques Cosnes, Silvio Danese, William J Sandborn, Maïté Lewin, Joel G Fletcher, Yehuda Chowers, Geert D'Haens, Brian G Feagan, Toshifumi Hibi, Daniel W Hommes, E Jan Irvine, Michael A Kamm, Edward V Loftus, Edouard Louis, Pierre Michetti, Pia Munkholm, Tom Oresland, Julian Panés, Laurent Peyrin-Biroulet, Walter Reinisch, Bruce E Sands, Juergen Schoelmerich, Stefan Schreiber, Herbert Tilg, Simon Travis, Gert van Assche, Maurizio Vecchi, Jean-Yves Mary, Jean-Frédéric Colombel, Marc Lémann

**Affiliations:** 1 Department of Hepatogastroenterology, Hôpital Saint-LouisParis, France; 2 Department of Gastroenterology and Nutrition, Hôpital Saint-AntoineParis, France; 3 Department of Gastroenterology, Instituto Clinico HumanitasRozzano, Milan, Italy; 4 Division of Gastroenterology, University of California San DiegoLa Jolla, California; 5 Department of Radiology, Hôpital Saint-AntoineParis, France; 6 Department of Radiology, Mayo ClinicRochester, Minnesota, USA; 7 Department of Gastroenterology, Rambam Health Care CampusBat Galim, Haifa, Israel; 8 Imelda GI Clinical Research CenterBonheiden, Belgium; 9 Robarts Research Institute, University of Western OntarioLondon, Ontario, Canada; 10 Division of Gastroenterology and Hepatology, Department of Internal Medicine, Keio University School of MedicineTokyo, Japan; 11 Department of Gastroenterology and Hepatology, Leiden University Medical CenterLeiden, The Netherlands; 12 University of Toronto and Division of Gastroenterology, St. Michael's HospitalToronto, Ontario, Canada; 13 StVincent's Hospital & University of MelbourneMelbourne, Australia; 14 Division of Gastroenterology and Hepatology, Mayo ClinicRochester, Minnesota, USA; 15 Department of Hepatogastroenterology, Centre Hospitalier Universitaire de Liège, Liège UniversityLiège, Belgium; 16 Department of Gastroenterology and Hepatology, Centre Hospitalier Universitaire Vaudois and University of LausanneLausanne, Switzerland; 17 Department of Medical Gastroenterology C, Herlev Hospital, University of CopenhagenDenmark; 18 Akershus University Hospital, Dept of GI Surgery, University in OsloNorway; 19 Gastroenterology Eepartment, Hospital Clinic of BarcelonaBarcelona, Spain; 20 Department of Hepatogastroenterology, Centre Hospitalier Universitaire de NancyVandoeuvre-Lès-Nancy, France; 21 Univ Klinik Innere Medizin IIIVienna, Austria; 22 MGH Crohn's and Colitis Center and Gastrointestinal Unit, Massachusetts General Hospital, Harvard Medical SchoolBoston, Massachusetts, USA; 23 CEA Hospital Board, University - Hospital Frankfurt/MainFrankfurt, Germany; 24 Institute of Clinical Molecular Biology, Christian-Albrechts UniversityKiel, Germany; 25 Christian Doppler Research Laboratory for Gut Inflammation, Medical University InnsbruckAustria; 26 Translational Gastroenterology Unit, John Radcliffe HospitalOxford, UK; 27 Division of Gastroenterology, University of Leuven HospitalsLeuven, Belgium; 28 Gastroenterology and Gastrointestinal Endoscopy Unit, IRCCS Policlinico San Donato & University of MilanSan Donato Milanese, Italy; 29 INSERM U717, Biostatistics and Clinical Epidemiology, Hôpital Saint-LouisParis, France; 30 Department of Hepatogastroenterology, Hôpital Huriez, Centre Hospitalier UniversitaireLille, France

**Keywords:** Crohn's disease, illness index severity, magnetic resonance imaging

## Abstract

Crohn's disease (CD) is a chronic progressive destructive disease. Currently available instruments measure disease activity at a specific point in time. An instrument to measure cumulative structural damage to the bowel, which may predict long-term disability, is needed. The aim of this article is to outline the methods to develop an instrument that can measure cumulative bowel damage. The project is being conducted by the International Program to develop New Indexes in Crohn's disease (IPNIC) group. This instrument, called the Crohn's Disease Digestive Damage Score (the Lémann score), should take into account damage location, severity, extent, progression, and reversibility, as measured by diagnostic imaging modalities and the history of surgical resection. It should not be “diagnostic modality driven”: for each lesion and location, a modality appropriate for the anatomic site (for example: computed tomography or magnetic resonance imaging enterography, and colonoscopy) will be used. A total of 24 centers from 15 countries will be involved in a cross-sectional study, which will include up to 240 patients with stratification according to disease location and duration. At least 120 additional patients will be included in the study to validate the score. The Lémann score is expected to be able to portray a patient's disease course on a double-axis graph, with time as the x-axis, bowel damage severity as the y-axis, and the slope of the line connecting data points as a measure of disease progression. This instrument could be used to assess the effect of various medical therapies on the progression of bowel damage. (Inflamm Bowel Dis 2011)

Crohn's disease (CD) is a chronic inflammatory bowel disorder which usually follows a relapsing and remitting course (flare-ups followed by clinical remission) in the early phases of the disease. Relapses are characterized by clinical symptoms associated with biological, endoscopic, and histological signs of inflammation. Even during periods of clinical remission, the bowel is not free of lesions; subclinical inflammation often persists and there is an evolution to fibrostenotic stricture or penetrating lesions (fistula and abscess) of the bowel, reflecting a progressive, destructive disease course in the later phases of the disease, resulting in structural bowel damage. Surgical resection of bowel is frequently required to treat strictures, fistula, or abscess, and it should be recognized that surgically resected bowel is the ultimate manifestation of bowel damage.^1^ Following surgery, this cycle often recurs, leading to progressive loss of intestinal function and disability.

Until now, the structural bowel damage component of CD has not been examined, with the therapeutic focus being on the assessment of disease activity judged by the severity of symptoms and inflammation. To assess the severity of clinical disease activity, composite scores such as the Crohn's Disease Activity Index (CDAI) or the Harvey–Bradshaw Index are used. To assess the severity of endoscopic inflammation, the Crohn's Disease Endoscopic Index of Severity (CDEIS), the Simplified Endoscopy Score (SES-CD), or, in the postoperative setting, the Rutgeerts' score were developed for use in clinical trials.^2^–^8^ These instruments estimate the severity of disease activity or inflammation and are currently the standard by which the efficacy of new products is assessed in clinical trials. While they can assess the severity of inflammatory activity at a specific timepoint, they do not gauge the cumulative structural bowel damage and thus do not capture the progressive, destructive course of the disease. For instance, CDAI and CDEIS scores can be similar in both patients with recent onset CD who are naïve to treatment and in patients with a long history of CD who have extensive, irreversible bowel damage from progressive inflammation or previous surgical resection.

The characterization of CD as a progressive, destructive disease is not new, but has become better recognized through longitudinal follow-up studies of large cohorts of patients with CD. Before these studies, CD was often regarded as a heterogeneous entity with different phenotypes. In 1998 the Vienna classification identified three subgroups of patients according to disease behavior: B1, purely inflammatory (nonpenetrating, nonstricturing); B2, fibrostenotic; and B3, penetrating.^9^ On this schematic classification, patients were assumed to show different patterns of evolution. In 2002, Louis et al^10^ and Cosnes et al^11^ independently demonstrated that most patients with CD actually had a nonpenetrating nonstricturing phenotype at diagnosis, but progressed to stricturing and penetrating lesions over the long term. They demonstrated that the natural history of CD is a dynamic process, leading to irreversible bowel damage in the large majority of patients. The findings of these referral center studies have recently been confirmed in a population-based cohort.^12^

The view of potential treatment goals in CD has also changed.^13^ The therapeutic objective is now evolving: it is not only to control disease activity in terms of clinical symptoms and inflammatory markers, but also to prevent progression of structural bowel damage. As in rheumatoid arthritis and other destructive inflammatory conditions, early intervention is being considered to prevent irreversible damage.^14^ A novel randomized trial compared two strategies, early combination immunosuppressive and anti-tumor necrosis factor (TNF) biologic treatment (top-down) versus a conventional step-up approach in patients recently diagnosed with CD.^15^ Although early intensive treatment accelerated clinical remission and limited corticosteroid exposure during the first year, the rate of clinical remission without steroids was similar for both strategies during the second year. These results can be interpreted in two contrasting ways: 1) early intervention was not more effective than the conservative strategy in preventing disease progression; 2) early intervention was effective, but the endpoints to assess disease progression were not appropriate. This result strongly suggests that long-term endpoints including assessment of bowel damage might demonstrate a benefit with early intensive treatment.

The concept of tissue damage has been explored extensively in rheumatoid arthritis. A specific damage index score measuring joint erosion and destruction over time using radiographs has been developed and is used to measure disease progression.^16^–^19^ Surface erosion and joint-space narrowing were selected as the most relevant criteria, because they occur frequently and are independent of each other. These two types of lesions are assessed on standard x-rays of hands, wrists, and feet. According to the severity of lesions in 28 joints graded on a semiquantitative scale, a score ranging from 0 to 448 is derived. Repeat X-ray can be used to assess the progression of joint damage.^19^ Since its development and validation, the modified Sharp index has been used in numerous clinical trials to compare various treatments and treatment strategies in patients with rheumatoid arthritis.^20^–^24^

The first bowel damage score for CD, proposed by Cosnes et al,^25^ was primarily a weighted cumulative measurement of surgically resected bowel. More recently, novel imaging methods, including abdominal endosonography, computed tomography enterography (CTE), and magnetic resonance imaging enterography (MRE), have provided accurate methods to identify structural bowel damage.^26^–^41^ These tools can therefore be used to develop an instrument analogous to the modified Sharp index to quantify damage, whether surgically resected bowel or bowel that remains in vivo. Such an instrument could measure disease progression over time and assess the impact of treatment strategies on the progression of CD.

The objective of this article is to describe the methods that will be used to develop this new instrument: the Crohn's Disease Digestive Damage Score (The Lémann score).

## METHODOLOGY OF THE DEVELOPMENT OF THE Lémann score

The International Program to develop New Indexes in Crohn's disease (IPNIC) group was formed in 2007.^42^ It is an international working group under the auspices of the French association INTESTINFO. It comprises 28 gastroenterologists from 15 countries, one surgeon, two radiologists, and one biostatistician. One of its objectives is to develop an instrument that can measure the cumulative bowel damage in patients with CD, the Lémann score.

### Expected Characteristics of the Lémann score

IPNIC group members have recommended that the Lémann score should be able to: 1) measure cumulative bowel damage at a specific time in a patient's history; 2) measure the progression of bowel damage over time in cohorts of patients and in clinical trials; 3) identify patients with CD at high (or low) risk of rapid damage progression; and 4) compare the effects of treatment on the progression of bowel damage to determine the responsiveness of the index.

The score should measure cumulative digestive tissue damage and be based on a comprehensive assessment of structural bowel damage, including stricturing lesions, penetrating lesions (fistulas and abscesses), and surgical resection. Its applicability should be broad and it should allow all patients to be assessed at different clinical stages (early or advanced CD, operated or nonoperated, with limited or extensive CD).

The assessment methods should be “damage driven” (i.e., based on damage location, extent, and severity, using appropriate diagnostic imaging modalities or a history of surgical resection) and not be “diagnostic modality driven.” The optimal diagnostic modalities may change with time and technical progress, but should be determined by the type of lesion and location (for example, CTE or MRE, and colonoscopy).

The index score should take into account damage location, extent, and severity. Damage location (upper digestive tract, small bowel, colon or rectum, and anal or perianal) is necessary to take into account the relative clinical importance of the location of the damage for progression (such as upper gastrointestinal disease) or outcome (such as perianal disease). To evaluate damage extent, the digestive tract will be divided into segments based on their clinical relevance, frequency of involvement, feasibility of defining limits to one given segment, and the Montreal Classification of disease.^43^ For each segment, severity will be scored on an ordinal scale ranging from 0 (normal) to 3 (maximal) for stricturing lesions, penetrating lesions, and surgical resection or bypass of bowel. As an example, Table 1 shows the severity grades proposed for small bowel stricturing or penetrating lesions and surgery or other interventional procedures. Weighting coefficients for individual items can be determined statistically or by expert consensus.

**TABLE 1:** Severity Scale for Small Bowel Lesions According to the Lesions or History of Surgery or Any Other Interventional Procedure

Grade	Stricturing Lesions	Penetrating Lesions	History of Surgery or Any Other Interventional Procedure
0	Normal	Normal	None
1	Wall thickening <3 mm and/or segmental enhancement without prestenotic dilatation	—	—
2	Wall thickening ≥3 mm and/or mural stratification without prestenotic dilatation	Deep transmural ulceration	Bypass diversion or stricturoplasty
3	Stricture with prestenotic dilatation	Abscess or any type of fistula	Resection

Finally, the score should vary from zero (no digestive damage) to a theoretical maximum value corresponding to complete resection of the digestive tract.

### Digestive Damage Assessment Methods

CTE and MRE have greatly improved the detection of structural small bowel lesions in CD.^26^–^41^ The high quality of images has made it possible to visualize precisely the location of lesions, bowel wall involvement, fat or mesenteric changes around segments of the gastrointestinal tract, and the presence of strictures, fistulas, or abscesses. A precise cartography of CD lesions is now possible.

#### Ultrasound

Ultrasound can be an informative imaging modality when performed by an experienced operator under ideal conditions. It provides an excellent view of the intestinal wall and can detect the presence of complications, particularly in ileal CD. In several studies its accuracy was comparable to that of MRE for evaluating wall thickening and disease activity.^44^ However, the use of ultrasound, particularly in clinical trials, is limited by the fact that it is highly operator-dependent, difficult to standardize examinations between centers, and difficult to acquire images that can be archived for serial comparisons over time. Central reading of ultrasound images is also challenging,^34^ because information is gained in real time. The ability of ultrasound to quantify the anatomic disease extent, particularly in small bowel CD, is therefore limited.

#### Computed Tomography Enterography

CTE has demonstrated over 80% sensitivity and specificity for detecting bowel segments affected by CD, as it allows multiplanar reformation with isovoxel resolution.^44^,^45^ In addition, CTE can easily be standardized, and images can be read centrally. The main drawback of CTE is the risk of repeated radiation exposure associated with the need for follow-up studies; recent publications have emphasized the potential risk of gastrointestinal cancer associated with repetitive use of abdominal computed tomography, especially in patients exposed at a young age.^45^ Even though this risk is low and theoretical, being based on extrapolations from the observed risk at higher exposure levels, it must be considered in CD, because the affected patient population is young and will require frequent reassessment.

#### Magnetic Resonance Imaging Enterography

MRE protocols used to assess bowel lesions in CD are similar to those used for CTE.^35^–^38^ MRE combines high-tissue-contrast examination with multiplanar acquisitions of the abdomen.^46^ Detection rates in publications with the latest generation of magnetic resonance imaging (MRI) equipment are generally similar to those achieved by CTE. A recent study underlined the accuracy of MRI for measuring disease activity in ileocolonic CD, with results comparable to colonoscopy.^39^ Nonetheless, access to MRI remains limited in some countries; image acquisition and analysis still takes longer than for CTE; and the value of colonic MRI needs to be validated by other centers. MRE has the potential to be the most useful imaging modality to evaluate bowel damage because of its accuracy, lack of ionizing radiation, and ability to detect penetrating complications of CD.

#### Upper Endoscopy and Colonoscopy

Upper gastrointestinal endoscopy and colonoscopy can identify mucosal lesions that more accurately reflect disease activity (inflammation) than bowel damage. The low frequency of the involvement of the upper tract in CD (<15%) is unlikely to justify routine upper endoscopy for the purposes of the present instrument.^47^ Colonoscopy, on the other hand, has been recommended for assessment of colonic damage because, despite improvements in MRI technique, biopsy confirmation of the nature of lesions and potential therapy can be performed with one technique. The inability of endoscopy or colonoscopy to evaluate lesions outside the bowel, as well as the interventional nature of examination often performed under sedation, are limitations of the techniques.

### Cross-sectional Study Aimed at Developing the Lémann score

The main objective of this multicenter, cross-sectional study is to develop an instrument that can measure the cumulative bowel damage at a specific point in time.

#### Study Design

Twenty-four centers in 15 countries will be involved in the collection of data. Each center will include at least one set of 10 patients (learning test set), and some centers will include an additional set of 10 patients (validation set). The Lémann score should allow patients to be assessed at different clinical stages (early or advanced CD, operated or nonoperated) and with four disease locations (upper gastrointestinal tract, small bowel, colon and/or rectum, and anal locations). Therefore, patients will be stratified within each center according to their present CD location and disease duration (<2 years, 2–10 years and ≥10 years) except for the upper gastrointestinal location, which is less frequent than the other CD locations. A target number of patients will be recruited into each stratum (Table 2).

**TABLE 2:** Patients Enrolled Into Each Set (Learning Test or Validation) in Each Center

Patient No.	CD Location^a^	CD Duration
1	Upper digestive tract	—b
2	Small bowel	< 2 years
3	Small bowel	[2-10] years
4	Small bowel	≥ 10 years
5	Colon and/or rectum	< 2 years
6	Colon and/or rectum	[2-10] years
7	Colon and/or rectum	≥ 10 years
8	Perianal and anal	< 2 years
9	Perianal and anal	[2-10] years
10	Perianal and anal	≥ 10 years

a Patient will have to present with at least the following CD location.

b Regardless of disease duration.

The final population will include up to 240 patients for the descriptive (learning test) set and a minimum of 120 patients for the validation set. If necessary, the validation set will be pooled with the descriptive (learning) set and bootstrap methods used to generate samples to evaluate the quality of the damage severity score, per disease location and globally.

To be included, patients should have abdominal MRE and pelvic MRI, upper endoscopy, and colonoscopy, according to their CD location (Table 3). If patients have abdominopelvic CTE, data will also be analyzed. MRI and CT data (if available) will be read by both the investigator and the investigative site radiologist.

**TABLE 3:** Examinations^a^ Required for Inclusion in the Study Aimed to Develop the Lémann score, According to Crohn's Disease Location

CD Location	UpperEndoscopy	Colonoscopy	Abdominal MRI Enterography	PelvicMRI	Abdominopelvic CT Enterography^b^
Upper digestive tract	X		X		X
Small bowel			X		X
Colon and/or rectum		X	X		X
Perianal and anal			X	X	X

CD, Crohn's disease; CT, computed tomography; MRI, magnetic resonance imaging.

a Additional examinations may be performed at the discretion of the investigator but are not required for inclusion in the study.

b CT enterography will be performed only in some patients.

Finally, for each CD location and globally for the whole gut the investigator will make an overall evaluation of damage severity on a visual analog scale (VAS) ranging from 0 (no damage) to 10 (complete destruction).

#### Data Analysis

##### Lémann score construction

The principle of the construction process will be to derive a score that is strongly correlated with the dependent variables: i.e., overall damage severity at each location and global damage severity for the whole digestive tract. This score will be based on independent ordinal variables describing lesions (strictures, penetration by ulcers, fistulas and abscess, and surgical resection of bowel) in each segment for the four CD locations. Bowel damage scoring will therefore be conducted in several steps.

In the first step, each location will be studied separately. For each CD location, independent variables will be coded as follows: (a) presence of a segment with at least grade 1, 2, or 3 lesion/surgery; (b) number of segments with at least grade 1, 2, or 3 lesion/surgery; (c) the proportion of segments containing grade 1, 2, or 3 lesion/surgery will also be assessed (if feasible) in the case of small bowel location; (d) only the presence of variables will be used in the case of anal location. The dependent variable will be the overall damage severity in the location as evaluated on a linear VAS. Multiple linear regression with both forward and backward selection procedures using the likelihood ratio test will be used to derive a location damage severity score as a linear combination of independent variables, describing presence and numbers or percentages of segments containing one type of lesion/surgery; this will be a combination that is strongly correlated with the dependent variable (overall damage severity in the CD location). For each CD location the score will be simplified as much as possible by rounding and grouping coefficients in the linear combination.

The second step is the analysis of data from all locations together. The aim of this second step is to test whether a common linear combination of independent variables could be applied to the different locations to predict the dependent variables. This procedure will use a mixed multiple linear model.

The third step will be to determine the weightings to be applied to each evaluation of overall damage severity in the various locations in calculating the global damage severity score. This will be performed by linear multiple regression, with the global evaluation as dependent variable and the four overall location damage severity evaluations as independent variables. The applicability of the model will be carefully checked. If the results of the third step are unsatisfactory, the investigators will determine a weighting by consensus. If the results obtained on the first and/or third steps seem encouraging, but could be improved by increasing sample size, the learning and validation test sets will be pooled to construct the index.

##### Lémann score validation

Results obtained in the first step, possibly simplified in the second step, will be validated by calculating the damage severity score within each location from data in the validation set. Parametric and nonparametric correlation coefficients between the calculated damage severity score and the damage severity assessment will be estimated, both by location and globally. If necessary, bootstrap methods will be used to generate new samples.

##### Complementary studies

The reproducibility of the MRI examination will be analyzed from the MRI recordings, with additional information from other sources (history of surgery, and results of complementary examinations such as colonoscopy, or clinical anal examination). Concordance between MRI and CT detection of strictures and penetrating lesions (fistulas and abscesses) will also be studied on a sample of patients with both MRI and CT data.

## DISCUSSION

The concept of CD as a progressive disease inducing cumulative structural damage has emerged over recent years.^10^–^12^ The present article describes the methodology for developing an instrument, called the Lémann score, which should enable assessment of cumulative structural bowel damage at a given time in a CD patient's history, taking into account both the extent and severity of bowel damage, including stricturing and penetrating lesions and previous surgery. Damage will be assessed based on the medical history, endoscopy, and other imaging techniques. It also offers the potential for evaluating the rate and progression of damage over a period of time through serial assessment. This should allow the effect of therapeutic intervention to be assessed.

For each lesion and location, the current optimal diagnostic imaging modality will be used. Imaging modalities are likely to evolve through technical progress. Of particular importance is that the number of tools used to construct the index should be as small as possible, to facilitate widespread use of the index. Ideally, a single investigation should be selected, MRE being a good candidate. However, access to abdominal and pelvic MRI is still limited, as may be radiological expertise, while the accuracy of MRE for assessment of the colon is still being evaluated. For the development of the Lémann score we have decided to include patients with additional investigations according to disease location in order to explore the information they could provide. More specifically, the usefulness of endoscopy has been debated: IPNIC group members decided that at this stage in the development of the score, colonoscopy is necessary for patients with a history of colonic involvement, to detect colonic strictures, and upper endoscopy for those known to have upper digestive tract disease. Pelvic MRI was also regarded as necessary in all patients with a history of perianal disease, whatever the results of clinical examination. It is also planned to include patients having both CT and MRI, to determine whether diagnostic modality may be used interchangeably.

**FIGURE 1:**
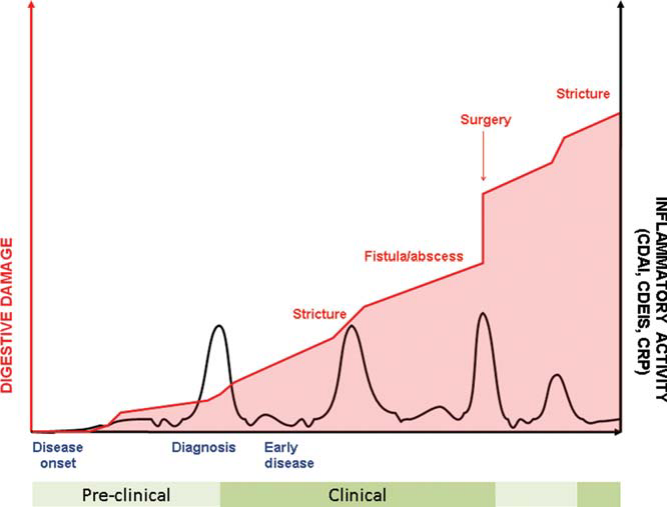
Progression of digestive damage and inflammatory activity in a theoretical patient with CD.

We expect that the Lémann score for a patient will be graphically represented on a double-axis graph, with time as the x-axis and bowel damage severity as the y-axis (Fig. 1). The location of the patient on the graph will describe cumulative disease damage at a specific point in time in the patient's history. At present, this information is intuitively taken into account by clinicians in therapeutic decision-making, but is not formally quantified. Damage severity is not taken into account in clinical trials or cohort studies for selection of patients or assessment of drug efficacy. The Montreal Classification provides a crude picture of damage, with location (L) and behavior (B) of the disease. For instance, a patient with limited ileal disease experiencing obstructive symptoms will be in the same category (L1, B2) as one with extensive small bowel disease, multiple strictures, and previous intestinal resection; obviously, therapeutic decisions may be different between these two patients. The Lémann score should provide a better measurement of the severity of structural bowel damage and may be used to measure bowel damage progression with repeated assessments. The slope of the curve of digestive damage could be taken into account for decision-making, independently of damage severity. As in rheumatoid arthritis, the slope of the curve may allow patients with rapid damage progression to be selected in order to propose intensified therapy, or to use in other cases less aggressive treatment. The effects of medical therapies or strategies on disease progression could also be evaluated.

The present article introduces the basis for the development of the CD digestive damage score, the Lémann score. Such a score should allow better identification of patients with severe damage and those with rapid progression of damage. The Lémann score has the potential to be integrated into clinical trials or prospective evaluation of cohorts of patients in the near future. In particular, justification for early intervention with immunosuppressive and/or biologic agents could be strengthened if an impact can be demonstrated on digestive disease damage.
